# Consequences of severe habitat fragmentation on density, genetics, and spatial capture-recapture analysis of a small bear population

**DOI:** 10.1371/journal.pone.0181849

**Published:** 2017-07-24

**Authors:** Sean M. Murphy, Ben C. Augustine, Wade A. Ulrey, Joseph M. Guthrie, Brian K. Scheick, J. Walter McCown, John J. Cox

**Affiliations:** 1 Department of Forestry, University of Kentucky, Lexington, Kentucky, United States of America; 2 Department of Fish and Wildlife Conservation, Virginia Polytechnic Institute and State University, Blacksburg, Virginia, United States of America; 3 Fish and Wildlife Research Institute, Florida Fish and Wildlife Conservation Commission, Gainesville, Florida, United States of America; Universidade Nova de Lisboa Instituto de Higiene e Medicina Tropical, PORTUGAL

## Abstract

Loss and fragmentation of natural habitats caused by human land uses have subdivided several formerly contiguous large carnivore populations into multiple small and often isolated subpopulations, which can reduce genetic variation and lead to precipitous population declines. Substantial habitat loss and fragmentation from urban development and agriculture expansion relegated the Highlands-Glades subpopulation (HGS) of Florida, USA, black bears (*Ursus americanus floridanus*) to prolonged isolation; increasing human land development is projected to cause ≥ 50% loss of remaining natural habitats occupied by the HGS in coming decades. We conducted a noninvasive genetic spatial capture-recapture study to quantitatively describe the degree of contemporary habitat fragmentation and investigate the consequences of habitat fragmentation on population density and genetics of the HGS. Remaining natural habitats sustaining the HGS were significantly more fragmented and patchier than those supporting Florida’s largest black bear subpopulation. Genetic diversity was low (*A*_R_ = 3.57; *H*_E_ = 0.49) and effective population size was small (*N*_E_ = 25 bears), both of which remained unchanged over a period spanning one bear generation despite evidence of some immigration. Subpopulation density (0.054 bear/km^2^) was among the lowest reported for black bears, was significantly female-biased, and corresponded to a subpopulation size of 98 bears in available habitat. Conserving remaining natural habitats in the area occupied by the small, genetically depauperate HGS, possibly through conservation easements and government land acquisition, is likely the most important immediate step to ensuring continued persistence of bears in this area. Our study also provides evidence that preferentially placing detectors (e.g., hair traps or cameras) primarily in quality habitat across fragmented landscapes poses a challenge to estimating density-habitat covariate relationships using spatial capture-recapture models. Because habitat fragmentation and loss are likely to increase in severity globally, further investigation of the influence of habitat fragmentation and detector placement on estimation of this relationship is warranted.

## Introduction

Primarily driven by anthropogenic activities, including expansion of urban development, agriculture, and transportation infrastructure, the loss and fragmentation of indigenous habitats have subdivided several previously contiguous large carnivore populations into multiple small and often isolated subpopulations [1–3]. Small populations are more vulnerable to genetic, demographic, and environmental stochasticity than their larger counterparts, which can result in heightened susceptibility to deleterious genetics effects (e.g., increased genetic drift and inbreeding depression) and cause precipitous population declines [4–6]. Immigration is needed for natural genetic and demographic rescue from these effects [7, 8], but such rescues are less likely to occur in areas where habitat loss and fragmentation have been severe [9].

Although the American black bear (*Ursus americanus*) is one of the most widely distributed large carnivores in North America, the species occupies a fraction of historical range in the southeastern United States [10, 11]. Within the Southeastern Coastal Plain, habitat loss and fragmentation have extirpated black bears from many areas and subdivided a once large, regional bear population into ≥ 13 individual subpopulations, many of which are small or isolated [11–14]. Seven subpopulations of the Florida black bear (*Ursus americanus floridanus*) subspecies are disjunctly distributed throughout the state of Florida, USA [15]. Indices suggested the Highlands-Glades subpopulation (HGS) in south-central Florida was the second smallest (*N* ≈ 150–200 bears [15]) among those 7 subpopulations, and an early 2000s genetic structure analysis indicated the HGS was isolated [12]. The HGS is the closest (~40 km) subpopulation to the southernmost Big Cypress subpopulation of Florida black bears, which faces potential threats of rising sea levels and increasing urban development [16]. Five other subpopulations occur ≥ 120 km north of the HGS, with large urban areas and an extensive road network, including the greater Orlando metropolitan area and Interstate-4 corridor, likely serving as formidable impediments to demographic and genetic connectivity with the HGS [12, 15]. Furthermore, lands supporting the HGS are predicted to lose ≥ 50% of remaining native habitats over the next 50 years [17], rendering the HGS deserving of higher conservation priority in one of the most biodiverse areas of North America [18]. Despite the potential for demographic and genetic consequences to worsen in the HGS from continued isolation caused by additional habitat loss, intensive monitoring has not occurred and little is known about this subpopulation [15].

Black bears are vagile habitat-generalists that are capable of long-range movements to overcome habitat fragmentation and other anthropogenic and natural landscape barriers [19]. Black bears also have considerable dietary plasticity and often exploit human-sourced foods if habitat availability is reduced [20], which can positively influence bear population vital rates [21]. Therefore, the species not only exhibits some resiliency to habitat fragmentation, but can thrive in mildly developed exurban areas [22]. The black bear is also iteroparous (i.e., overlapping generations), a reproductive strategy that can slow the development of deleterious genetics effects associated with isolation of small populations if sufficient habitat is available to support population growth [23]. Such ecological and biological elasticity has caused difficulty in research aimed at quantifying the demographic and genetic consequences of habitat fragmentation on black bear populations [12, 24, 25].

Density is an invaluable demographic parameter because it can be compared across wildlife populations of varying abundance and geographic distribution to provide insight into ecological relationships and associated conservation implications [26]. Spatial capture-recapture models directly estimate population density based on the spatial distribution of detections, estimating the probability of detection as a function of distance between detectors and animal activity centers (i.e., centroid of the space that an individual occupies [27–29]). Recent extensions to spatial capture-recapture models, including the incorporation of habitat and landscape covariates in the density model (i.e., spatially inhomogeneous density), can improve estimate accuracy and provide information about salient ecological relationships [22, 30–35]. The use of these models to quantify wildlife population density-habitat relationships and inform landscape connectivity is expected to increase in the future as fragmentation and loss of native habitats intensifies in many regions of the world [36].

Given the lack of critical ecological information for the conservation of HGS black bears and the threat of impending habitat fragmentation and loss, we used noninvasive genetic sampling in a multi-year spatial capture-recapture study to evaluate the consequences of habitat fragmentation on density and population genetics of this subpopulation. Our primary objectives were to: 1) quantify the degree of habitat fragmentation for lands supporting the HGS; 2) estimate HGS density and abundance to provide critical baseline estimates for conservation; and 3) investigate temporal changes in genetic diversity and effective population size of the HGS. We hypothesized that: 1) habitat fragmentation would be more severe than that of an area supporting Florida’s largest black bear subpopulation; 2) population density of the HGS would be low and abundance would be smaller than previously presumed via indices; and 3) genetic diversity and effective population size of the HGS would remain relatively constant over time.

## Materials and methods

### Study area

**Fig 1:**
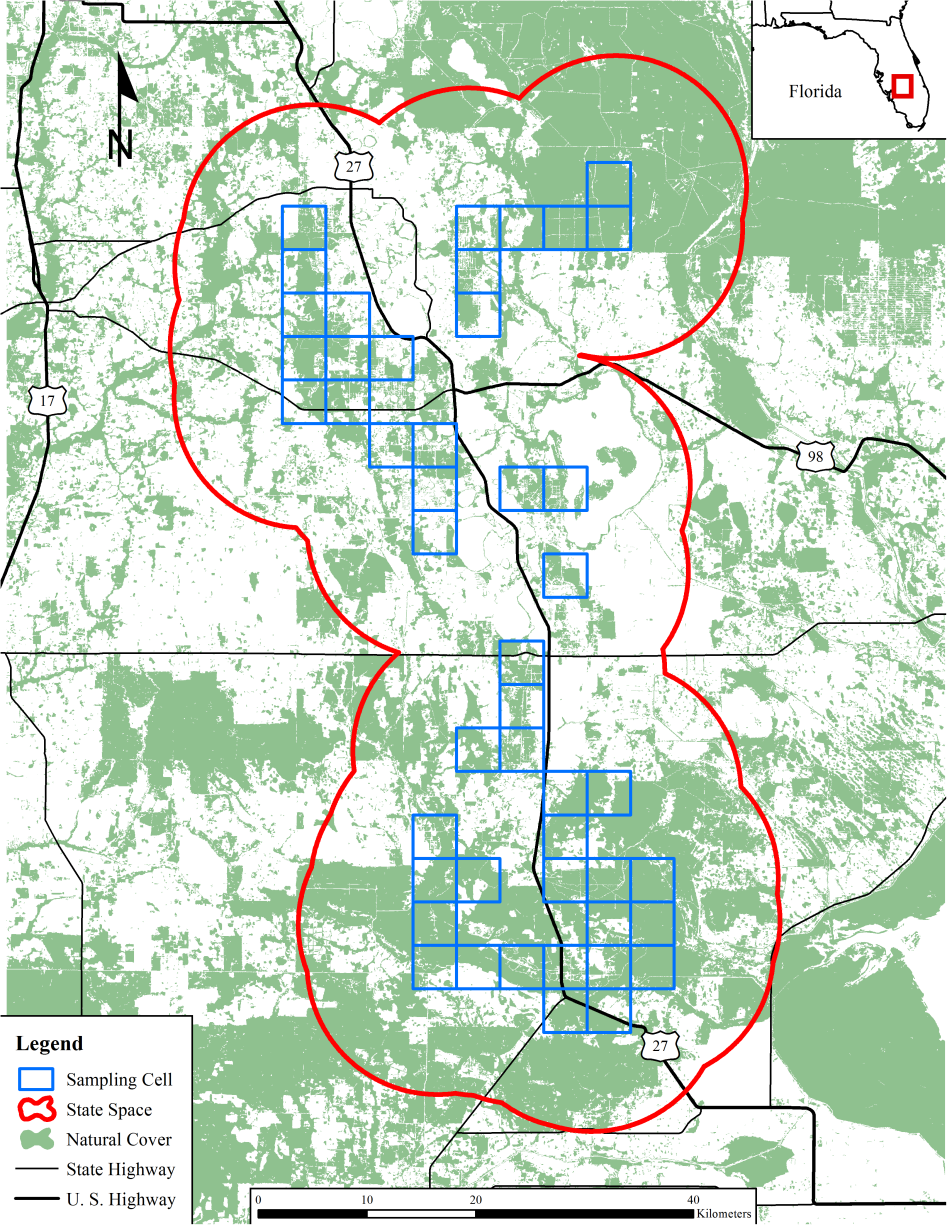
Locations of 46 sampling cells in south-central Florida, USA, in which a single, baited barbed-wire hair trap was placed in each cell to collect black bear hair for estimating density and population genetics of the Highlands-Glades subpopulation of Florida black bears (2010–2012).

The study area was located in south-central Florida, USA, approximately midway between the Atlantic Ocean and the Gulf of Mexico (Fig 1). The climate was humid sub-tropical with hot, wet summers and mild, dry winters. Average annual precipitation was 136 cm and average annual temperature was 21.2°C [37]. The study area was located at the southern terminus of the endangered Lake Wales Ridge ecosystem, with an average elevation of 47 m above sea level [38]. Numerous freshwater insular lakes were scattered throughout the study area; the largest was Lake Istokpoga (112.07 km^2^). Sandy, nutrient-poor soils support multiple xeric upland habitat communities, including federally endangered scrub [39], scrubby flatwoods, and sandhills. Margins of the Lake Wales Ridge and surrounding lands are a mosaic of mesic and hydric habitats that include pine (*Pinus sp*.) flatwoods, hardwood hammocks, and dry prairies, and bayheads, freshwater marshes, and bald cypress (*Taxodium distichum*) swamps, respectively [38]. The study area was bisected by U.S. Highway 27, a major thoroughfare running North–South the entire length of Florida. Agriculture was the dominant land cover type in the area, primarily plantations of citrus monocultures and cattle ranches. Human population density averaged 21 people/km^2^, and the largest city was Sebring (10,331 people [40]).

### Sampling

We collected black bear hair from barbed-wire hair traps [41] in an 8-occasion, 3-session capture-recapture study during 2010–2012. We used a trap spacing of 4.0 km × 4.0 km (16.0-km^2^ area/trap), which corresponded to 2 traps per estimated average annual HGS female bear home range size (32.17 km^2^ [42]), and established a 2,016 km^2^ sampling grid comprised of 126 contiguous sampling cells using ArcMap 10.3 (ESRI, Redlands, California, USA). However, we only placed hair traps in sampling cells for which ≥ 20% of the cell’s area was comprised of natural cover (i.e., black bear habitat [15]); this resulted in a single, baited hair trap in each of 46 sampling cells (36.5% of cells; Fig 1). We did not construct traps within citrus groves, which are human-created monocultures that provide traversing cover for bears, but are not considered primary or secondary habitat and do not offer a primary food for bears [43–45]. We constructed all traps using 2 wires placed at 25 and 50 cm above the ground; both were wrapped around 3–5 trees to create an approximately 25-m^2^ enclosure. We baited hair traps with pastries and raspberry scent lure, hung from a string approximately 2 m above the ground in the center of the trap. We checked and re-baited traps for a total of 8, 7-day sampling occasions, which occurred in blocks of 2 consecutive occasions followed by a 7-day break, during May–October of each year. We did not move traps between sampling occasions, but we did move traps between sessions (i.e., years). We treated each barb as an individual sample and removed hairs using tweezers that were sterilized between sample collections using flame from a lighter. We stored hair samples in individually labeled paper coin envelopes and used a flame to sterilize barbs after sample collections to eliminate spurious captures.

We also acquired a dataset of HGS black bear hair samples that were collected during 2004–2005 from live-captures, mortalities, and opportunistic noninvasive methods (e.g., from barbed-wire fencerows). These samples were representative of the HGS-specific cohort that was used to characterize genetic structure of all 7 Florida black bear subpopulations [12], but were not the identical samples used in that study. We used these samples to investigate temporal changes in population genetics parameters that are important to conservation of small populations (e.g., genetic diversity and effective population size [25, 46, 47]).

All sampling methods were approved by a University of Kentucky Institutional Animal Care and Use Committee (Protocol #00626A2003) and occurred under an approved Florida Fish and Wildlife Conservation Commission Scientific Research Permit (#LSSC-10-00112A). No threatened or endangered species were involved in this study. Sampling on privately owned lands, including those for which conservation easements were established, occurred with explicit permission from individual landowners. The Nature Conservancy issued permission for sampling on all lands managed under the institution’s authority; Florida Park Service granted permission for sampling at Highlands Hammock State Park; United States Air Force issued permission for sampling at Avon Park Air Force Range; United States Fish and Wildlife Service issued permission for sampling at Lake Wales Ridge National Wildlife Refuge; and Florida Fish and Wildlife Conservation Commission granted permission for sampling at Fisheating Creek Wildlife Management Area, and Lake Wales Ridge and Platt Branch Wildlife and Environmental Areas.

### Laboratory analyses

All collected hair samples were sent to Wildlife Genetics International (Nelson, British Columbia, Canada) for DNA extraction and amplification using the standard protocols described by Paetkau [48]. For financial reasons, we subsampled each year’s collected samples in the 2010–2012 data, but not the 2004–2005 data, by selecting one sample per trap per occasion for genotyping during each occasion. This subsampling protocol results in nominal estimate bias (< 2.50%) and adequate confidence interval coverage (≥ 0.96) of true population density if population sizes are small (≤ 500 individuals) and spatially fixed-density spatial capture-recapture models are used [31]. To perform the subsampling, laboratory personnel randomized the samples collected from each trap during each occasion (i.e., trap-occasion) and selected the first sample from each trap containing ≥ 20 underfur hairs or ≥ 5 guard hair roots for genotyping, repeating this process for each trap-occasion. If no samples within a trap-occasion met this threshold, laboratory personnel chose the next best available sample using a minimum quality threshold of 5 underfur hairs or 1 guard hair root. Standard protocols were used for DNA extraction [48], a sex marker was used to determine sex of each individual, and the following 12 microsatellite markers were used to identify individuals [49]: G1A, G1D, G10B, G10C, G10H, G10J, G10L, G10M, G10P, G10X, MU50, and MU59. To minimize genotyping error and reduce the chances of inflating the number of individuals because of this error, the methods described by Paetkau [48] were used.

### Population genetics

We used MICRO-CHECKER v2.2.3 [50] to test for genotyping errors, including scoring errors, allelic dropout, and null alleles. We estimated probability of identity (*PI*), or the probability that 2 individuals have the same genotype, and probability of siblings (*PI*_Sibs_), or the probability that 2 individuals are related [51], using GENEALEX v6.5 [52]. We tested for Hardy-Weinberg equilibrium between genotypes and quantified linkage disequilibrium with *P*-values adjusted using Bonferroni correction [53] in GENEPOP v4.6 [54]. We estimated allelic richness (*A*_R_) via rarefaction to account for sample size discrepancies, expected (*H*_E_) and observed (*H*_O_) heterozygosity, and inbreeding coefficient (*F*_IS_) using the R software [55] package diveRsity [56]. We calculated 95% confidence intervals using 1,000 bootstrap iterations and considered non-overlapping confidence intervals between parameter estimates for the 2 sampling periods as significantly different.

We followed the methods described by Waples et al. [57] to estimate the effective number of breeders (*N*_B_; i.e., the number of individuals that reproductively contributed to the population) using the linkage disequilibrium method in N_E_ESTIMATOR v2.01 [58], with a minimum allele frequency of 0.05. We corrected for bias in *N*_B_ caused by the overlapping generations of bears and estimated effective population size (*N*_E_) using the adjustment formulas developed by Waples et al. [57]. These formulas reduce estimate bias to ≤ 5% for iteroparous species by incorporating 2 life history traits that explain the majority of variation in *N*_E_ [57, 59, 60]: age at sexual maturity (α; i.e., primiparity) and adult life span (AL). For example, early sexual maturity, long life span, and constant fecundity with age tend to produce *N*_B_/*N*_E_ ratios > 1, whereas delayed maturity, short life span, and variable fecundity with age produce ratios < 1 [57]. The incorporation of AL results in estimates that correspond to the ages at which a species is reproductively active, because juveniles are not effective breeders and thus, cannot contribute to *N*_B_. We used α = 4 years, based on the average age of primiparity for Florida black bears [61], and a maximum age of 24 years [15] to calculate AL of 21 years (i.e., AL = maximum age–α + 1). We corrected 95% confidence intervals of *N*_B_ and *N*_E_ by applying the same adjustment formulas to the lower and upper bounds.

To search for evidence of a genetic bottleneck, we evaluated departure from mutation-drift equilibrium using BOTTLENECK v1.2.02 [62]. We used a stepwise mutation model (SMM) and a two-phase model (TPM) that incorporated 30% of multi-step mutations to account for uncertainties in the mutation process [25, 63]. We performed 10,000 replications and assessed for a bottleneck using a Wilcoxon sign-rank test [64–66]. We performed factorial correspondence analysis using GENETIX v4.05 [67] to identify genetic substructure (i.e., > 1 genetic cluster) within the HGS as an indicator of incoming gene flow (i.e., immigration). Factorial correspondence analysis uses multivariate categorical data to identify structural relationships without requiring prior information, such as the presumed potential number of genetic clusters [68]. This method was well-suited for our data because the lack of genetics information for the other 6 Florida black bear subpopulations during 2010–2012 precluded more extensive analyses of genetic structure and migration [69]. We created a 2-dimensional plot to reflect the correspondence between individuals and allele combinations, estimated the total inertia (i.e., overall variation) present among individuals, and investigated potential immigration based on deviations from identified genetic clusters [70, 71].

### Demographics

We fit spatial capture-recapture models using maximum likelihood implemented in the R package secr [72] to estimate population density (*D*). We used a binomial observation model with a half-normal detection function, and modeled hair traps as proximity detectors because an individual could be detected at multiple traps during a single occasion [29, 73]. Simulations demonstrated that modeling traps as proximity detectors was appropriate for the subsampling protocol that we used [31]. We used the suggest.buffer function in the secr package to identify the appropriate state space, or the distance around traps within which all individuals that could have potentially been detected were included [29].

We created a habitat covariate that we modeled on *D* to attempt to improve estimate accuracy by predicting *D* to covariate values where traps did not exist [22, 30, 34]. Using 2011 National Land Cover Database data with 30-m resolution [74] and ArcMap 10.3, we reclassified deciduous, evergreen, and mixed forests, woody and emergent herbaceous wetlands, and shrub-scrub as natural cover; and developed, barren land, grassland, pasture-hay, and cultivated crops as non-natural cover. We then created a percent natural cover covariate (Pnat) using the Geomorphometry and Gradient Metrics Toolbox v2.0 [75] to smooth the reclassified raster and calculate percent cover within each 30-m raster cell using a moving circular window with a 3.20-km radius, which corresponded to the radius of estimated average annual female home range size [42, 76].

We developed a set of a priori sex-specific models that included expected sources of variation in detection function parameters based on previous black bear hair trapping studies in the eastern and southeastern United States that also used spatial capture-recapture models for analysis of multi-session sampling data [31, 76–78]. To account for individual heterogeneity in detection probability, we considered 2-class finite mixtures (h2) on the probability of detection at the activity center of an individual (*g*_*0*_ [79, 80]); we did not use individual covariates [81] because individual-based ancillary data for hair samples did not exist. Because we baited hair traps, we included a trap specific-behavioral response (bk) on *g*_*0*_ in all models [31, 82]. We fixed (~1) the spatial scale of the detection function (σ) and fixed *D*, allowed *D* to vary among sessions (i.e., years [Y]) as a factor, or allowed *D* to vary with Pnat following a log-linear relationship.

We evaluated models with Akaike’s Information Criterion corrected for small sample size (AIC_*c*_) and produced sex-specific estimates of *D*, *g*_*0*_, and σ [83]. We considered all models ≤ 2 ΔAIC_*c*_ competitive, reverting to the most parsimonious model for parameter estimation if fit of competing models was not an improvement over the top model [84]. To investigate if male to female sex ratios differed significantly, we evaluated deviation from 1:1 based on the absence of 95% confidence interval overlap of sex-specific *D* estimates. We derived abundance (*N*) from *D* as the expected number of individuals within available natural cover in the state space [85]. We produced total population *D* and *N* by adding sex-specific estimates and obtaining the combined variances, assuming independence [86]. We only produced estimates for natural cover within the state space because negligible occurrence data for bears on lands outside of the state space precluded informed extrapolation.

### Habitat fragmentation

We used FRAGSTATS v4.2.1.603 [87] to evaluate habitat fragmentation (i.e., contagion) within the state space and estimate percent land area that was natural cover, patch density, and mean patch size [88]; contagion ranges from 0 to 100%, with 0 indicating maximal fragmentation. We used the natural versus non-natural cover raster (see Demographics subsection) and defined natural patches using the eight neighbor rule [87]. We compared the resulting values to those produced by Hostetler et al. [89] for the Ocala-St. Johns subpopulation of Florida black bears. This provides an informative comparison because the Ocala-St. Johns subpopulation is relatively large [78] and is sustained almost entirely by large blocks of federally managed forested lands [15], whereas the HGS inhabits mostly privately owned lands that are vulnerable to anthropogenic development [90, 91].

## Results

### Population genetics

During 2004–2005, 72 black bear hair samples were collected, 51 (71%) of which assigned to 34 (17M:17F) individuals; 21 (29%) samples failed genotyping. We collected 1,484 samples during the 2010–2012 capture-recapture hair trapping; 455 (31%) samples were selected for genotyping via our subsampling protocol, but 159 of those failed during analysis. From the remaining 296 (20%) samples, we identified 74 (33M:41F) unique individuals; 11 (4M:7F) individuals were also present in the 2004–2005 data. Annual detections were 33 (10M:23F), 33 (14M:19F), and 48 (22M:26F) bears during 2010, 2011, and 2012, respectively.

Scoring errors and allelic dropout were not present in the 2004–2005 data, but evidence of a null allele was detected at locus G10M. In contrast, we found no evidence of scoring errors, allelic dropout, or null alleles in the 2010–2012 data. Probability of identity (*PI*) was 1.0^−5^ and 8.2^−7^ for the 2004–2005 and 2010–2012 data, respectively, and *PI*_Sibs_ was 4.1^−3^ and 1.5^−3^, respectively. The criteria for HWE were met in both datasets (2004–2005: $\chi_{22}^{2}$ = 35.09, *P* = 0.04; 2010–2012: $\chi_{24}^{2}$ = 33.87, *P* = 0.09) following Bonferroni correction (α < 0.002). Non-random association of alleles was detected at 11% and 8% of 66 pairwise comparisons in the 2004–2005 and 2010–2012 data, respectively, after applying Bonferroni correction (α < 0.0007). Because no consistent patterns of null alleles, HWE deviation, or linkage disequilibrium were present at identical loci between the 2 datasets, we did not exclude any loci from analyses [92].

**Fig 2:**
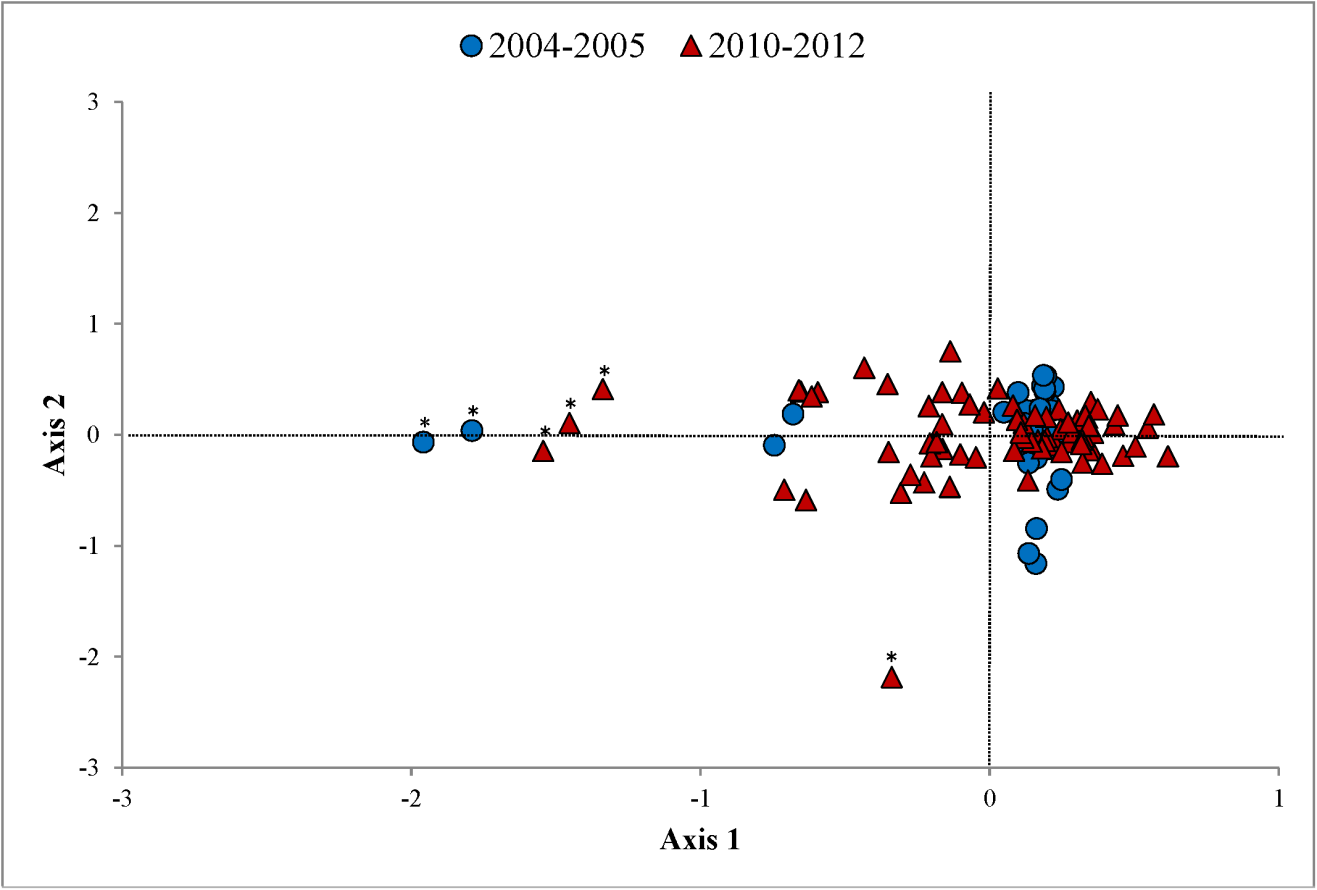
Two-dimensional plot depicting the correspondence between individuals and allele combinations for Florida black bears in the Highlands-Glades subpopulation of south-central Florida, USA. We estimated inertia (overall variation) detected during 2004–2005 and 2010–2012 from factorial correspondence analysis. Potential immigrants identified are indicated by asterisks (*).

We did not find significant differences in population genetics parameter estimates between the 2 time periods (Table 1). All measures of genetic diversity (*A*_R_, *H*_E_, and *H*_O_) and effective sizes (*N*_B_ and *N*_E_) were low, but no evidence of inbreeding (*F*_IS_) was detected. A genetic bottleneck was not supported in the 2004–2005 data (SMM: *P* = 0.88; TPM: *P* = 0.06), but we did find weak support in the 2010–2012 data (SMM: *P* = 0.92; TPM: *P* = 0.03). Factorial correspondence analysis indicated that 37.29% and 22.34% of inertia existed among individuals in the 2004–2005 and 2010–2012 data, respectively. No substructuring was present in the HGS during either period, but 2 and 4 potential immigrants were detected during 2004–2005 and 2010–2012, respectively (Fig 2).

**Table 1:** Temporal comparison of genetics parameter estimates for the Highlands-Glades subpopulation of Florida black bears in south-central Florida, USA, based on 12 microsatellite markers. We estimated allelic richness (*A*_R_), observed heterozygosity (*H*_O_), expected heterozygosity (*H*_E_), effective number of breeders (*N*_B_), effective population size (*N*_E_), and inbreeding coefficient (*F*_IS_) from 12 microsatellites for bears sampled during 2004–2005 and 2010–2012. Confidence intervals (95%) are presented in parentheses and *n* corresponds to sample size.

Period	*n*	Parameter					
		*A*_R_	*H*_O_	*H*_E_	*N*_B_	*N*_E_	*F*_IS_
**2004–2005**	34	3.02 (2.44–3.61)	0.39 (0.28–0.50)	0.42 (0.30–0.54)	7 (4–11)	14 (8–24)	0.07 (**–**0.03–0.17)
**2010–2012**	74	3.57 (3.06–4.08)	0.50 (0.41–0.60)	0.49 (0.40–0.58)	12 (9–16)	25 (19–33)	**–**0.02 (**–**0.08–0.04)

### Demographic estimates

The optimal buffer distance around trap locations was 12 km with 1-km point spacing, which we used as the state space. There were 2 competing (≤ 2 ΔAIC_*c*_) models for females, both of which included *D* spatially varying by the Pnat covariate (Table 2). Four models were competing for males; the second-ranked model suggested *D* varied among years, the third-ranked model included *D* varying by the Pnat covariate, and the first and fourth-ranked models both indicated spatially homogenous (i.e., fixed) *D*.

**Table 2:** Spatial capture-recapture models for estimating density of female and male Florida black bears in the Highlands-Glades subpopulation of south-central Florida, USA (2010–2012). We modeled percent natural cover (Pnat) as a habitat covariate on density (*D*), allowed *D* to vary among sessions (Y), or fixed (~1) *D*. We modeled a trap-specific behavioral response (bk) and 2-class finite mixtures (h2) on the probability of detection at the activity center of an individual (*g*_*0*_), and fixed the spatial scale of the detection function (σ). Model selection was based on ≤ 2 ΔAIC_*c*_, which is the relative difference between AIC_*c*_ (Akaike’s Information Criterion corrected for small sample size) of the model and the highest ranked model. Weight (w_i_) and log-likelihood (logLik) are presented for each model.

Model	# Parameters	AIC_*c*_	ΔAIC_*c*_	w_i_	logLik
Females					
*D*(~Pnat) *g*_*0*_(~bk) σ(~1)	5	1077.21	0.00	0.69	–533.12
*D*(~Pnat) *g*_*0*_(~bk + h2) σ(~1)	7	1078.83	1.62	0.31	–531.48
*D*(~1) *g*_*0*_(~bk) σ(~1)	4	1088.20	10.99	0.00	–539.78
*D*(~1) *g*_*0*_(~bk + h2) σ(~1)	6	1089.14	11.93	0.00	–537.88
*D*(~Y) *g*_*0*_(~bk) σ(~1)	6	1091.85	14.64	0.00	–539.23
*D*(~Y) *g*_*0*_(~bk + h2) σ(~1)	8	1093.10	15.89	0.00	–537.33
Males					
*D*(~1) *g*_*0*_(~bk) σ(~1)	4	898.26	0.00	0.30	**–**444.64
*D*(~Y) *g*_*0*_(~bk) σ(~1)	6	898.65	0.39	0.25	**–**442.25
*D*(~Pnat) *g*_*0*_(~bk) σ(~1)	5	899.23	0.97	0.18	**–**443.86
*D*(~1) *g*_*0*_(~bk + h2) σ(~1)	6	899.93	1.67	0.13	**–**442.89
*D*(~Y) *g*_*0*_(~bk + h2) σ(~1)	8	900.88	2.62	0.08	**–**440.49
*D*(~Pnat) *g*_*0*_(~bk + h2) σ(~1)	7	901.47	3.22	0.06	**–**442.26

**Fig 3:**
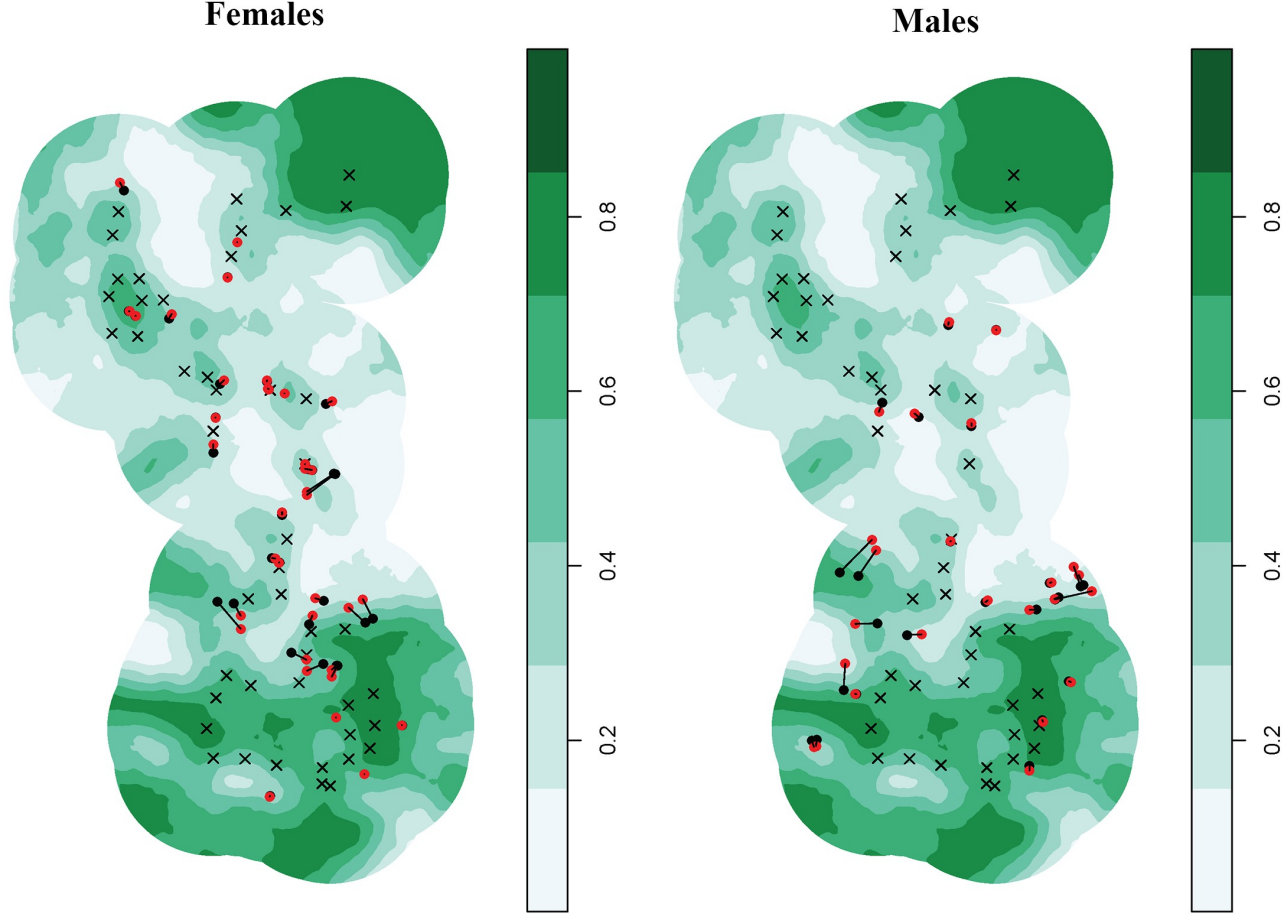
Sex-specific posterior modes of activity centers estimated by spatially inhomogeneous and homogenous density spatial capture-recapture models relative to percent natural cover for Florida black bears in the Highlands-Glades subpopulation of south-central Florida, USA (2010–2012). Posterior modes from inhomogeneous (varies by percent natural cover) and homogenous density models are indicated by red and black circles, respectively. Locational shifts for each posterior mode between models are denoted by solid black lines, and crosses (**×**) represent the 46 hair traps that were established. Locations where only a red circle is visible without a solid black connector line indicates a black circle is at the same location. Percent natural cover within the state space is the background color gradient from white (low %) to dark green (high %).

Spatially inhomogeneous *D* models produced negative coefficient estimates for Pnat (i.e., density decreased with increasing percent cover; Table 3). Upon further inspection, we discovered that the locations of posterior modes of activity centers estimated by homogenous *D* models were on the periphery of the trap array in medium to high percent cover (≥ 40%; Fig 3). In contrast, several posterior modes of activity centers estimated by the inhomogeneous *D* models were shifted further away from the trap array and into low percent cover (< 0.20), which led to relatively large changes in the covariate values associated with those locations. This was consistent with a negative *D*-covariate relationship, but also indicated misspecification of other components of the model that could push some activity centers to non-habitat. Because of this uncertainty and possible ecologically implausible *D*-covariate relationship, we removed the inhomogeneous *D* models from further consideration (see Discussion for further commentary).

**Table 3:** Spatial capture-recapture model coefficient estimates (β) from the top spatially inhomogeneous density model for male and female Florida black bears in the Highlands-Glades subpopulation of south-central Florida, USA (2010–2012). Model structure included density (*D*) varying with percent natural cover (Pnat), a trap-specific behavioral response (bk) on the probability of detection at the activity center of an individual (*g*_*0*_), and fixed spatial scale of the detection function (σ). Estimate standard errors (SE) and lower (LCL) and upper (UCL) 95% confidence limits are presented.

Parameter	Females				Males			
	β	SE	LCL	UCL	β	SE	LCL	UCL
***D***	–7.24	0.36	–7.96	–6.53	–8.92	0.50	–9.89	–7.95
***D*~Pnat**	–2.58	0.76	–4.07	–1.10	–1.37	1.17	–3.66	0.93
***g***_***0***_	–1.87	0.34	–2.54	–1.19	–3.49	0.29	–4.05	–2.93
***g***_***0***_**~bk**	1.52	0.36	0.81	2.22	2.12	0.34	1.46	2.79
**σ**	7.85	0.10	7.65	8.05	8.97	0.11	8.75	9.18

Among the remaining models, the highest ranked spatially homogenous *D* model for each sex included bk on *g*_*0*_ and fixed σ, which we produced estimates from. The combined population *D* was 0.054 bear/km^2^ (95% CI = 0.041–0.067), which was significantly female-biased and corresponded to *N* of 98 (95% CI = 75–122) total bears in available habitat (Table 4). The spatial scale of the detection function (σ) was approximately 3 times larger for males, whereas *g*_*0*_ was 5 times higher for females (Table 4).

**Table 4:** Sex-specific spatial capture-recapture model parameter estimates for the Highlands-Glades subpopulation of Florida black bears in south-central Florida, USA (2010–2012). We estimated the following parameters, averaged over 3 years of sampling: the probability of detection at the activity center of an individual (*g*_*0*_), the spatial scale of the detection function (σ [km]), and density (*D* [bear/km^2^]). We derived abundance (*N*) from *D* and confidence intervals (95%) are presented in parentheses.

Parameter	Males	Females	Highlands-Glades
***g***_***0***_	0.025 (0.013–0.047)	0.126 (0.076–0.203)	
**σ**	8.098 (6.146–10.672)	2.626 (2.166–3.184)	
***D***	0.015 (0.010–0.022)	0.039 (0.028–0.052)	0.054 (0.041–0.067)
***N***	27 (18–40)	71 (51–95)	98 (75–122)

### Habitat fragmentation

Fragmentation metrics revealed that the 1,827.09 km^2^ of natural cover (i.e., bear habitat) within the state space was severely fragmented with a patchier distribution than lands supporting the larger Ocala-St. Johns subpopulation of Florida black bears (Table 5). Percent land area that was natural habitat and mean patch size were lower and patch density higher than lands occupied by the Ocala-St. Johns subpopulation. Contagion was considerably lower than that for the Ocala-St. Johns subpopulation, indicating patches of natural cover in the area occupied by the HGS were disaggregated and more dispersed.

**Table 5:** Habitat fragmentation metrics estimated for natural habitats supporting the Highlands-Glades and Ocala-St. Johns subpopulations of Florida black bears. We estimated percent land area that was natural habitat (% HLA), patch density (PD; patches/km^2^), mean patch size (MPS; km^2^), and contagion (Contag; %) for lands occupied by the HGS, and compared to values produced by Hostetler et al. [89] for lands occupied by the comparatively larger Ocala-St. Johns subpopulation.

Subpopulation	% HLA	PD	MPS	Contag
**Highlands-Glades**	47.74	2.97	0.34	32.54
**Ocala-St. Johns**	89.30	0.15	5.80	92.00

## Discussion

Habitat fragmentation and loss can isolate wildlife populations and have severe consequences on their demographics and genetics, but those effects can be difficult to identify in wide-ranging large carnivores that have long generation times, such as bears. We collected genetics data via noninvasive sampling from Florida black bears in a subpopulation that was previously identified as being isolated and presumed to be small as a result of habitat fragmentation and loss. Estimates of genetic diversity for the HGS (Table 1) remain significantly lower than estimates for large black bear populations that resided in relatively contiguous habitats (e.g., *H*_E_ > 0.70; *A*_R_ > 6.00 [25, 92–94]), and were comparable to populations that suffered isolation-induced bottlenecks [24, 25, 49, 95, 96]. Although a sample size discrepancy existed between the 2 periods for which we estimated genetics parameters, simulations demonstrated that *H*_E_ is unaffected by differing sample sizes, as is *A*_R_ if estimated via rarefaction as we did [97]. We found weak support for a genetic bottleneck in the HGS, but detection power from highly variable microsatellites is typically poor (≤ 0.27) if samples are collected ≥ 10 generations after a bottleneck occurs [63]. Indeed, habitat loss to agriculture and urban development in south-central Florida began escalating during the 1920s [91], suggesting the bottleneck may have occurred nearly a century prior to our study, or approximately 15 generations based on the average generation time of black bears (~6.3 years [98, 99]).

Although one migrant per generation has been adopted as a general rule for preventing genetic deterioration in wild populations [100], ≥ 3 migrants are typically needed to increase variation and positively influence population fitness, particularly if the *N*_E_/*N* ratio is ≤ 0.2 [101]. For example, Seal and Lacy [102] predicted that 8 mountain lions from Texas, USA (*Puma concolor stanleyana*) would need to be introduced to the genetically degraded Florida panther (*Puma concolor coryi*) population to increase genetic diversity by 20% [103, 104]. Effective population size (*N*_E_) and *F*_IS_, both of which are indicators of genetic fitness [47], were unchanged in the HGS between sampling periods despite the identification of 3–4 potential, although unconfirmed, immigrants in the 2010–2012 data (Table 1; Fig 2). Given that the duration between sampling periods encompassed one complete black bear generation, and considering the *N*_E_/*N* ratio for the HGS (0.25), 3 migrants per generation may be the minimum necessary to prevent the loss of, but not increase, genetic variation in the HGS.

Obtaining reliable, accurate estimates of population abundance and density is fundamental for informing conservation of genetically depauperate wildlife populations. We considered spatially inhomogeneous density (*D*) spatial capture-recapture models that allowed *D* to vary with habitat covariate values to attempt to improve estimate accuracy in the portions of the state space where we did not place traps [30]. Although models that included the percent natural cover covariate (Pnat) were the most parsimonious for female bears and were among competing models for males (Table 2), coefficient estimates for the *D*-Pnat relationship were negative and predicted that *D* was highest in areas with the lowest percentages of natural cover (< 20%; Table 3). Sollmann et al. [33] found a similar inverse relationship between black bear population *D* and percent cover using spatial capture-recapture models with resource selection functions; however, the lowest percent cover in that study was 62%, whereas a substantial portion of lands in the HGS study area had 0% natural cover (Fig 3). Although black bears are habitat generalists that can be synanthropic in areas of low anthropogenic development along the wildland-urban interface (i.e., exurban areas [22]), that cities or large expanses of open ranchlands present in the study area could solely support or be preferentially selected by an entire bear population as suggested by the inhomogeneous *D* models is unlikely. Corn (*Zea mays*) dispensed at remote wildlife feeders by white-tailed deer (*Odocoileus virginianus*) hunters in areas of high quality natural cover was the dominant human-sourced food item in the diet of HGS bears across all seasons, whereas garbage comprised < 1% of consumed foods [45]. Furthermore, few radio-monitored HGS bears frequented urban developments [42, 43], collectively indicating that most HGS bears are probably not residing in areas of low or no natural cover.

The black bear populations of interest in other studies that evaluated spatially inhomogeneous *D* models inhabited landscapes comprised of relatively contiguous habitats [22, 31, 34]. The HGS, however, resides in habitat that is severely fragmented and has a considerably higher patch density and smaller mean patch size than lands supporting Florida’s largest black bear subpopulation (Table 4 [89]), which occupies the greatest expanse of protected areas among all 7 subpopulations [15]. The locations of many posterior modes of activity centers estimated by the top spatially homogenous *D* models were on the periphery of the trap array, which is also where Pnat began declining; however, the spatially inhomogeneous *D* models moved several of those posterior modes to areas of low percent cover (< 20%; Fig 3). Even slight model misspecification, such as the presence of individual heterogeneity in *g*_*0*_ or σ [105], can incorrectly place activity centers further away from traps, which may mimic a negative *D*-covariate relationship in severely fragmented landscapes where traps are only placed in moderate to high quality habitat.

The combination of severe habitat fragmentation and hair traps being placed only in areas of moderate to high percent natural cover may have rendered the spatially inhomogeneous *D* models unable to accommodate other model misspecification. For example, misspecification of the functional form of the *D*-covariate relationship or not including important covariates [33, 34], neither of which could we investigate because of low power and the absence of traps in areas presumed to be poor bear habitat. Indeed, natural cover is unlikely to comprehensively describe suitable black bear habitat, as other covariates, such as distance to roads and human population densities, can also influence bear space use [22, 106, 107]. Natural cover does, however, provide an informative generalization when population-specific habitat use information is unavailable, which is often the case for small or otherwise imperiled populations of bears and other carnivores (e.g., [108]).

Nonetheless, establishing hair traps in non-habitat where presumably no bears reside (e.g., urban developments and open ranchlands) would probably improve specification of the *D*-covariate relationship; however, bear researchers will likely be reluctant to implement hair trap sampling in non-habitat for logistical, financial, and social reasons. In contrast, deploying remote cameras in non-habitat and modeling photo detections versus non-detections as occupancy data [109] within a spatially explicit framework using models similar to those developed by Chandler and Clark [110] would be a feasible and statistically reasonable alternative. Regardless, we caution that spatially inhomogeneous *D* models appear to be sensitive to misspecification of the *D*-covariate relationship if suitable habitats are severely fragmented and the trap array does not sample the entire range of covariate values. Considering habitat loss and fragmentation are likely to increase globally commensurate with projected human population growth [111], which may increase the use of spatially inhomogeneous *D* models for estimating wildlife populations that inhabit fragmented landscapes [27, 36], further investigation of this issue via simulation is warranted. Additionally, the hair subsampling protocol that we used results in reliable *D* estimates for bear populations identified as having spatially homogenous *D* [31]. Whether this is true for populations with spatially varying *D* is unclear, however, because the loss of critical spatial recaptures to subsampling that could have placed activity centers in moderate to high percent natural cover may degrade model reliability; thus, further investigation of the effects of subsampling is also needed (e.g., [82, 112]).

Given the probably erroneous predictions of the spatially inhomogeneous *D* models, we estimated model parameters using the next most supported sex-specific models in which *D* was spatially fixed (Table 2). Estimated *D* for the HGS (0.054 bear/km^2^) had high precision (coefficient of variation = 0.124) and was significantly female-biased, but approached the lowest estimated via spatial capture-recapture models for black bear populations in the United States (lowest: 0.040–0.046 bear/km^2^ [77, 78]). Our estimate corresponded to *N* of 98 (95% CI: 75–122) total bears in available habitat, or a 53–104% smaller population size than previously presumed and for which conservation and management decisions have been made [15]. The absence of previous *D* and *N* estimates for the HGS precluded an evaluation of long-term temporal demographic changes, but comparing estimates of *N*_E_ between 2004–2005 and 2010–2012 suggests *D* and *N* have probably remained relatively constant over time.

In large bear populations, sex ratios skewed towards females are typically indicative of population growth and expansion [31, 113, 114]. Considering the small size of the HGS and the severity of habitat fragmentation, however, breeding opportunities may have been reduced [115, 116] by relatively high male bear mortality from anthropogenic causes compared to male abundance (*n* = 8 male bear deaths/year [117], or 30% of *N*_Male_ annually). Although anecdotal, the female-biased sex ratio, low genetic diversity, *N*_E_ smaller than necessary for long-term viability [47], small *N*, and low *D* are collectively indicative of a population that is potentially on the verge of deteriorating into an extinction vortex if habitat fragmentation and loss continue as projected [118]. For example, Palomares et al. [119] discovered that a female-biased sex ratio and low genetic diversity were among the factors contributing to an extinction vortex in a similarly small, low density, and isolated population of another terrestrial carnivore, the Iberian lynx (*Lynx pardinus*). Given the substantial loss of natural habitats in south-central Florida that is expected in coming decades, which could further reduce population size and erode genetic variation, efforts to preserve remaining lands comprised of natural habitats, possibly by acquiring conservation easements or government ownership, will likely be critical to long-term persistence of the HGS. Additionally, implementing a program to monitor population vital rates (e.g., survival and reproductive rates) and genetics of the HGS would allow modeling subpopulation growth and genetic diversity over time [15]. Such a program would provide more conclusive information on the status and potential future of this small subpopulation of bears that is faced with imminent deleterious landscape changes [120, 121].

## Supporting information

S1 Appendix Data, microsatellite genotypes for individual Florida black bears detected in the Highlands-Glades subpopulation via noninvasive genetic capture-recapture hair trap sampling during 2004–2005 and 2010–2012. (DOCX)

S2 Appendix Data, noninvasive genetic capture-recapture hair trap detections and corresponding trap locations for Florida black bears in the Highlands-Glades subpopulation during 2010–2012. (DOCX)
