# Supplementary material for: Consequences of severe habitat fragmentation on density, genetics, and spatial capture-recapture analysis of a small bear population
Source: PLoS One. 2017 Jul 24;12(7):e0181849. doi: 10.1371/journal.pone.0181849 (PMC5524351; doi:10.1371/journal.pone.0181849)
Supplement: S1 Appendix — (DOCX) [file pone.0181849.s001.docx]

**S1 Appendix. Data, microsatellite genotypes for individual Florida black bears detected in the Highlands-Glades subpopulation via noninvasive genetic capture-recapture hair trap sampling during 2004–2005 and 2010–2012.**

Table A. Individual bears (rows) were genotyped at the following 12 microsatellite loci during 2004–2005: G1A, G1D, G10B, G10C, G10H, G10J, G10L, G10M, G10P, G10X, MU50, and MU59.

| **2004–2005** | | | | | | | | | | | | |
| --- | --- | --- | --- | --- | --- | --- | --- | --- | --- | --- | --- | --- |
| **Sex** | **G1A** | **G10B** | **G10C** | **G1D** | **G10H** | **G10J** | **G10M** | **G10P** | **G10L** | **G10X** | **MU50** | **MU59** |
| F | 190194 | 152156 | 207213 | 172176 | 249253 | 187203 | 212214 | 159163 | 139139 | 141149 | 122134 | 239243 |
| M | 190198 | 152156 | 207215 | 176176 | 255255 | 199203 | 212212 | 147159 | 139139 | 141149 | 122122 | 243243 |
| M | 190198 | 152156 | 215215 | 176184 | 241255 | 203203 | 210210 | 147163 | 139139 | 141141 | 122122 | 243243 |
| F | 190190 | 152156 | 215215 | 184184 | 241253 | 199203 | 210212 | 163163 | 139139 | 141141 | 122122 | 243243 |
| M | 198198 | 156156 | 207215 | 184184 | 241253 | 203203 | 210212 | 163163 | 139139 | 141141 | 122122 | 239243 |
| F | 190198 | 152156 | 207215 | 176176 | 253253 | 203203 | 210212 | 159163 | 139139 | 141141 | 122122 | 243243 |
| F | 196198 | 152156 | 207215 | 176176 | 253255 | 203203 | 210210 | 163163 | 139139 | 141141 | 122122 | 243243 |
| F | 190190 | 152156 | 215215 | 176178 | 241253 | 203203 | 210210 | 163163 | 139139 | 141149 | 122122 | 243243 |
| M | 190198 | 152156 | 215215 | 176184 | 255255 | 203203 | 212212 | 159163 | 139139 | 141141 | 122122 | 243243 |
| M | 198198 | 156156 | 207215 | 176184 | 241241 | 203203 | 212212 | 161163 | 139139 | 141149 | 122122 | 243243 |
| F | 196198 | 156156 | 207207 | 176184 | 241241 | 185203 | 210210 | 147161 | 139139 | 149149 | 122122 | 239243 |
| F | 190198 | 152156 | 207215 | 184184 | 255255 | 199203 | 212212 | 159163 | 139139 | 141141 | 122122 | 243243 |
| F | 190198 | 156156 | 207215 | 184184 | 253255 | 199203 | 210210 | 163163 | 139139 | 141141 | 122122 | 243243 |
| F | 196198 | 152152 | 207215 | 184184 | 255255 | 199203 | 210210 | 161163 | 139139 | 141149 | 122122 | 243243 |
| F | 190198 | 152156 | 215215 | 176176 | 255255 | 203203 | 212212 | 163163 | 139139 | 141141 | 122122 | 243243 |
| F | 198198 | 152156 | 215219 | 184184 | 241255 | 203203 | 210212 | 163163 | 139139 | 141141 | 122122 | 239243 |
| F | 196198 | 152152 | 207215 | 176184 | 255255 | 203203 | 210210 | 161163 | 139139 | 141141 | 122122 | 243243 |
| F | 190190 | 156156 | 215215 | 184184 | 255255 | 199203 | 212212 | 159163 | 139139 | 141141 | 122122 | 243243 |
| F | 198198 | 156156 | 215215 | 176184 | 241241 | 203203 | 212212 | 161163 | 139139 | 141149 | 122122 | 243243 |
| F | 190198 | 156156 | 215215 | 176176 | 255255 | 203203 | 212212 | 159163 | 139139 | 141149 | 122122 | 243243 |
| F | 190190 | 156156 | 215215 | 184184 | 255255 | 199203 | 210210 | 163163 | 139139 | 141141 | 122122 | 243243 |
| F | 190198 | 156156 | 215215 | 176176 | 253255 | 199203 | 210212 | 159163 | 139139 | 141141 | 122122 | 243243 |
| M | 196198 | 152152 | 207207 | 184184 | 241255 | 185199 | 210210 | 161163 | 139139 | 141149 | 122122 | 239243 |
| M | 198200 | 152156 | 215215 | 172176 | 253255 | 187199 | 210212 | 163163 | 139139 | 141149 | 122134 | 243243 |
| M | 190190 | 156164 | 207215 | 172176 | 249253 | 187199 | 214214 | 161163 | 139139 | 141141 | 122134 | 239243 |
| M | 190190 | 156156 | 215215 | 176184 | 255255 | 203203 | 212212 | 163163 | 139139 | 141149 | 122122 | 243243 |
| M | 198198 | 152152 | 207215 | 176184 | 241253 | 199203 | 210212 | 159163 | 139139 | 141141 | 122122 | 243243 |
| M | 190198 | 152156 | 215215 | 176184 | 253255 | 199203 | 210210 | 147163 | 139139 | 141141 | 122122 | 243243 |
| M | 190198 | 156156 | 207215 | 176176 | 241241 | 203203 | 212212 | 163163 | 139139 | 149149 | 122122 | 243243 |
| M | 190198 | 156156 | 215215 | 176176 | 241241 | 203203 | 212212 | 163163 | 139139 | 141149 | 122122 | 243243 |
| M | 194198 | 152156 | 215215 | 172176 | 241253 | 187199 | 210212 | 163163 | 139139 | 141149 | 122126 | 239243 |
| M | 190198 | 152156 | 207207 | 176184 | 253255 | 203203 | 210210 | 161163 | 139139 | 141141 | 122122 | 243243 |
| M | 196198 | 156156 | 207215 | 176184 | 241255 | 185203 | 210210 | 161163 | 139139 | 141149 | 122122 | 239243 |
| M | 190190 | 156156 | 215215 | 184184 | 241255 | 199203 | 212212 | 159159 | 139139 | 141141 | 122122 | 243243 |

Table B. Individual bears (rows) were genotyped at the following 12 microsatellite loci during 2010–2012: G1A, G1D, G10B, G10C, G10H, G10J, G10L, G10M, G10P, G10X, MU50, and MU59.

| **2010–2012** | | | | | | | | | | | | |
| --- | --- | --- | --- | --- | --- | --- | --- | --- | --- | --- | --- | --- |
| **Sex** | **G1A** | **G10B** | **G10C** | **G1D** | **G10H** | **G10J** | **G10M** | **G10P** | **G10L** | **G10X** | **MU50** | **MU59** |
| F | 190190 | 152156 | 215215 | 176176 | 253255 | 199203 | 210212 | 159163 | 139139 | 141141 | 122122 | 243243 |
| M | 190190 | 156160 | 207215 | 176184 | 241255 | 187199 | 212212 | 159163 | 139139 | 141149 | 122122 | 239243 |
| F | 198198 | 152156 | 207207 | 184184 | 241255 | 199203 | 212212 | 159161 | 139139 | 141141 | 122122 | 243243 |
| F | 190198 | 152152 | 215215 | 176176 | 241255 | 185203 | 210212 | 163163 | 139139 | 141141 | 122122 | 243243 |
| F | 190198 | 156156 | 215215 | 184184 | 241255 | 199203 | 210212 | 163163 | 139139 | 141149 | 122122 | 243243 |
| F | 190198 | 154156 | 207215 | 176184 | 241255 | 187203 | 212214 | 159163 | 139155 | 149149 | 122122 | 239243 |
| F | 190190 | 156156 | 215215 | 184184 | 255255 | 199203 | 212212 | 159163 | 139139 | 141141 | 122122 | 243243 |
| F | 196198 | 152156 | 207215 | 176176 | 253255 | 203203 | 210210 | 163163 | 139139 | 141141 | 122122 | 243243 |
| M | 196198 | 156156 | 207215 | 176184 | 241255 | 185203 | 210210 | 161163 | 139139 | 141149 | 122122 | 239243 |
| M | 190190 | 156156 | 215215 | 176176 | 255255 | 199203 | 212212 | 159159 | 139139 | 141149 | 122122 | 239243 |
| F | 190198 | 156156 | 215215 | 184184 | 241255 | 203203 | 210212 | 163163 | 139139 | 141141 | 122122 | 243243 |
| M | 196198 | 152156 | 215215 | 176184 | 241255 | 203203 | 210212 | 159161 | 139139 | 141141 | 122122 | 239243 |
| M | 198198 | 156156 | 215215 | 184184 | 251255 | 185203 | 210210 | 161163 | 139139 | 141149 | 122122 | 243243 |
| F | 190198 | 152156 | 215215 | 176176 | 241253 | 203203 | 210212 | 163163 | 139139 | 141141 | 122122 | 243243 |
| M | 190190 | 152156 | 215215 | 176184 | 241241 | 203203 | 210212 | 163163 | 139139 | 141141 | 122122 | 243243 |
| M | 194198 | 156156 | 213215 | 172176 | 241249 | 203203 | 212212 | 163163 | 139139 | 141149 | 122134 | 239243 |
| F | 190200 | 152154 | 207215 | 176184 | 241253 | 187199 | 210214 | 163163 | 139155 | 149149 | 122134 | 243243 |
| M | 190198 | 156156 | 207215 | 176176 | 241241 | 203203 | 210212 | 147163 | 139139 | 141149 | 122122 | 243243 |
| M | 190190 | 156156 | 215215 | 176184 | 241253 | 199203 | 210214 | 163163 | 139139 | 141141 | 122134 | 243243 |
| F | 194198 | 152156 | 207215 | 176178 | 241255 | 199203 | 210210 | 159163 | 139139 | 141149 | 122126 | 243243 |
| M | 190200 | 154156 | 207215 | 176184 | 253253 | 185187 | 212214 | 147163 | 139139 | 141149 | 122134 | 239243 |
| F | 190190 | 156160 | 215215 | 184186 | 253255 | 187203 | 210212 | 147163 | 135139 | 141149 | 122122 | 243243 |
| F | 190198 | 152156 | 207215 | 176176 | 253255 | 199199 | 212212 | 159159 | 139139 | 141141 | 122122 | 239243 |
| F | 196198 | 156156 | 215215 | 172176 | 241253 | 187203 | 210212 | 163163 | 139139 | 141141 | 122122 | 239243 |
| F | 190196 | 156164 | 207215 | 176176 | 253255 | 187203 | 210214 | 163163 | 139139 | 141141 | 122134 | 239243 |
| M | 190190 | 154164 | 207207 | 184186 | 241241 | 185199 | 210214 | 159161 | 139139 | 147149 | 122134 | 239243 |
| F | 190198 | 156160 | 215215 | 176184 | 241241 | 187203 | 212212 | 159163 | 139139 | 141149 | 122134 | 239243 |
| F | 190190 | 156160 | 207215 | 176176 | 241253 | 199203 | 210212 | 159159 | 139155 | 141149 | 122122 | 239243 |
| F | 190190 | 156160 | 215215 | 176176 | 241253 | 187203 | 210214 | 159163 | 139139 | 141149 | 122134 | 239243 |
| M | 190200 | 156156 | 207215 | 176184 | 253253 | 187199 | 212214 | 163163 | 157157 | 149149 | 126126 | 239243 |
| M | 190198 | 156156 | 207207 | 176184 | 241255 | 199203 | 210212 | 159163 | 139139 | 141141 | 122122 | 239243 |
| F | 190198 | 152156 | 207215 | 176176 | 253255 | 203203 | 212212 | 159163 | 139139 | 141141 | 122122 | 243243 |
| M | 190198 | 156156 | 207215 | 176176 | 241255 | 199203 | 210212 | 159163 | 139139 | 141149 | 122122 | 239243 |
| F | 190198 | 156156 | 207215 | 176176 | 253255 | 199203 | 210212 | 159163 | 139139 | 141141 | 122122 | 243243 |
| F | 190198 | 156156 | 207215 | 176184 | 253255 | 185199 | 210212 | 159161 | 139139 | 141141 | 122122 | 243243 |
| M | 190198 | 156156 | 207207 | 176176 | 241255 | 199203 | 210212 | 159163 | 139139 | 141149 | 122122 | 239243 |
| M | 190198 | 156156 | 215215 | 176184 | 241255 | 187203 | 212212 | 159159 | 139139 | 141149 | 122122 | 239243 |
| M | 198200 | 156160 | 215215 | 172172 | 241241 | 187187 | 212212 | 163163 | 139155 | 141141 | 122134 | 235239 |
| M | 196200 | 156160 | 215215 | 176176 | 253253 | 187203 | 210212 | 163163 | 139139 | 141141 | 122126 | 239239 |
| F | 190198 | 156156 | 207207 | 176184 | 255255 | 203203 | 210210 | 161163 | 139139 | 141141 | 122122 | 243243 |
| M | 190200 | 156160 | 207215 | 172176 | 241253 | 187199 | 212212 | 147163 | 139155 | 141149 | 126134 | 235239 |
| M | 190198 | 152156 | 215215 | 172176 | 241253 | 199203 | 210212 | 159163 | 139139 | 141149 | 122122 | 239243 |
| F | 198198 | 152156 | 215215 | 176176 | 241253 | 187203 | 212212 | 163163 | 139139 | 141149 | 122122 | 243243 |
| F | 190198 | 156160 | 215215 | 176184 | 255255 | 187203 | 210212 | 163163 | 139139 | 141149 | 122122 | 243243 |
| M | 198200 | 152160 | 207215 | 172176 | 241253 | 187203 | 210212 | 163163 | 139139 | 141149 | 122126 | 239243 |
| M | 194198 | 152156 | 207215 | 176178 | 241253 | 187199 | 210210 | 163163 | 139139 | 149149 | 122126 | 239243 |
| F | 194198 | 152156 | 207215 | 176176 | 241249 | 187203 | 212214 | 159163 | 139139 | 141141 | 122122 | 243243 |
| F | 190194 | 152156 | 207213 | 172176 | 249253 | 187203 | 212214 | 159163 | 139139 | 141149 | 122134 | 239243 |
| F | 190190 | 152156 | 215215 | 176176 | 241253 | 203203 | 210212 | 163163 | 139139 | 141141 | 122122 | 243243 |
| F | 196198 | 152156 | 207215 | 172176 | 253253 | 187203 | 210212 | 163163 | 139139 | 141149 | 122122 | 239243 |
| M | 194198 | 152152 | 215215 | 172178 | 253255 | 187203 | 210210 | 163163 | 139139 | 141149 | 122122 | 239243 |
| M | 190198 | 156156 | 207215 | 176176 | 241241 | 203203 | 212212 | 163163 | 139139 | 149149 | 122122 | 243243 |
| M | 198198 | 152156 | 215215 | 176184 | 241253 | 199203 | 210210 | 163163 | 139139 | 141141 | 122134 | 239243 |
| M | 190198 | 154156 | 207215 | 176184 | 253253 | 185203 | 210212 | 147159 | 139139 | 141141 | 122122 | 243243 |
| F | 190198 | 152152 | 215215 | 176186 | 253255 | 187203 | 210210 | 159163 | 135139 | 141141 | 122122 | 243243 |
| M | 198198 | 152156 | 215215 | 176184 | 255255 | 203203 | 210212 | 159161 | 139139 | 141149 | 122122 | 243243 |
| F | 198198 | 156156 | 215215 | 176184 | 253255 | 203203 | 210212 | 159163 | 139139 | 141149 | 122122 | 239243 |
| F | 198198 | 152152 | 215215 | 176176 | 253255 | 199203 | 210210 | 147159 | 139139 | 141141 | 122122 | 243243 |
| M | 194198 | 156156 | 207215 | 176176 | 241253 | 185187 | 210210 | 161163 | 139139 | 141149 | 122126 | 239243 |
| F | 190198 | 154156 | 207215 | 176184 | 241241 | 187203 | 212214 | 159163 | 139139 | 141149 | 122122 | 239243 |
| F | 190190 | 156156 | 215215 | 176176 | 241253 | 187203 | 212214 | 163163 | 139139 | 141149 | 122122 | 239243 |
| M | 198200 | 152152 | 215215 | 176184 | 253255 | 187203 | 212212 | 163163 | 139139 | 141141 | 122134 | 239243 |
| F | 190198 | 156156 | 207215 | 184184 | 241255 | 199203 | 212212 | 159159 | 139139 | 141149 | 122122 | 243243 |
| F | 198198 | 156156 | 215215 | 176184 | 241241 | 203203 | 212212 | 161163 | 139139 | 141149 | 122122 | 243243 |
| F | 198198 | 152156 | 215215 | 176176 | 255255 | 199199 | 210210 | 159163 | 139139 | 141141 | 122122 | 243243 |
| M | 190198 | 152156 | 207215 | 176176 | 241241 | 185203 | 210210 | 147163 | 139139 | 149149 | 122122 | 239243 |
| F | 190194 | 156160 | 215215 | 172186 | 253255 | 187199 | 210212 | 163163 | 135139 | 141141 | 122126 | 239243 |
| M | 198198 | 156156 | 207215 | 176176 | 241253 | 199203 | 212212 | 159163 | 139139 | 141149 | 122122 | 239243 |
| M | 198198 | 152152 | 207215 | 176176 | 253255 | 185203 | 210210 | 163163 | 139139 | 141149 | 122122 | 243243 |
| F | 190190 | 156160 | 207215 | 176184 | 241255 | 199199 | 212214 | 163163 | 139139 | 141149 | 122134 | 239243 |
| F | 190198 | 156156 | 215215 | 176176 | 255255 | 203203 | 212212 | 159163 | 139139 | 141149 | 122122 | 243243 |
| F | 196198 | 152152 | 207215 | 176184 | 255255 | 203203 | 210210 | 161163 | 139139 | 141141 | 122122 | 243243 |
| F | 190198 | 156156 | 207215 | 184184 | 253255 | 199203 | 210210 | 163163 | 139139 | 141141 | 122122 | 243243 |
| M | 198198 | 156156 | 207215 | 176184 | 241241 | 203203 | 212212 | 161163 | 139139 | 141149 | 122122 | 243243 |
