# Supplementary material for: Consequences of severe habitat fragmentation on density, genetics, and spatial capture-recapture analysis of a small bear population
Source: PLoS One. 2017 Jul 24;12(7):e0181849. doi: 10.1371/journal.pone.0181849 (PMC5524351; doi:10.1371/journal.pone.0181849)
Supplement: S2 Appendix — (DOCX) [file pone.0181849.s002.docx]

**S2 Appendix. Data, noninvasive genetic capture recapture hair trap detections and corresponding trap locations for Florida black bears in the Highlands-Glades subpopulation during 2010–2012.**

Table A. Individual detection events for all bears detected across 8 occasions during each of 3 sessions (i.e., years).

| **Session** | **Bear ID** | **Sex** | **Occasion** | **Trap** |
| --- | --- | --- | --- | --- |
| 2010 | 14 | F | 2 | 157 |
| 2010 | 39 | M | 2 | 183 |
| 2010 | F12 | F | 1 | 140 |
| 2010 | F12 | F | 3 | 140 |
| 2010 | F12 | F | 4 | 140 |
| 2010 | F12 | F | 7 | 140 |
| 2010 | F16 | F | 2 | 158 |
| 2010 | F28 | F | 1 | 90 |
| 2010 | F28 | F | 2 | 89 |
| 2010 | F28 | F | 2 | 90 |
| 2010 | F28 | F | 5 | 89 |
| 2010 | F28 | F | 8 | 89 |
| 2010 | F30 | F | 4 | 136 |
| 2010 | F5 | F | 2 | 109 |
| 2010 | F5 | F | 6 | 136 |
| 2010 | F5 | F | 8 | 135 |
| 2010 | F5 | F | 8 | 136 |
| 2010 | F7 | F | 8 | 163 |
| 2010 | HG10-020 | F | 3 | 43 |
| 2010 | HG10-020 | F | 4 | 17 |
| 2010 | HG10-020 | F | 5 | 17 |
| 2010 | HG10-020 | F | 5 | 42 |
| 2010 | HG10-020 | F | 6 | 18 |
| 2010 | HG10-020 | F | 7 | 43 |
| 2010 | HG10-020 | F | 8 | 43 |
| 2010 | HG10-020 | F | 8 | 44 |
| 2010 | HG10-030 | M | 5 | 81 |
| 2010 | HG10-030 | M | 6 | 81 |
| 2010 | HG10-030 | M | 7 | 81 |
| 2010 | HG10-041 | F | 2 | 109 |
| 2010 | HG10-042 | M | 3 | 109 |
| 2010 | HG10-043 | F | 4 | 109 |
| 2010 | HG10-043 | F | 7 | 109 |
| 2010 | HG10-052 | M | 2 | 140 |
| 2010 | HG10-052 | M | 5 | 165 |
| 2010 | HG10-052 | M | 8 | 119 |
| 2010 | HG10-062 | F | 5 | 140 |
| 2010 | HG10-062 | F | 8 | 140 |
| 2010 | HG10-067 | M | 3 | 205 |
| 2010 | HG10-067 | M | 5 | 179 |
| 2010 | HG10-067 | M | 8 | 153 |
| 2010 | HG10-067 | M | 8 | 179 |
| 2010 | HG10-067 | M | 8 | 205 |
| 2010 | HG10-068 | F | 1 | 157 |
| 2010 | HG10-070 | F | 3 | 157 |
| 2010 | HG10-070 | F | 6 | 157 |
| 2010 | HG10-077 | F | 7 | 158 |
| 2010 | HG10-077 | F | 8 | 158 |
| 2010 | HG10-079 | F | 1 | 163 |
| 2010 | HG10-079 | F | 4 | 163 |
| 2010 | HG10-079 | F | 7 | 163 |
| 2010 | HG10-080 | F | 2 | 163 |
| 2010 | HG10-093 | F | 1 | 179 |
| 2010 | HG10-093 | F | 6 | 181 |
| 2010 | HG10-093 | F | 7 | 181 |
| 2010 | HG10-096 | F | 6 | 179 |
| 2010 | HG10-096 | F | 7 | 179 |
| 2010 | HG10-099 | M | 1 | 181 |
| 2010 | HG10-099 | M | 5 | 181 |
| 2010 | HG10-099 | M | 5 | 205 |
| 2010 | HG10-099 | M | 7 | 205 |
| 2010 | HG10-099 | M | 8 | 181 |
| 2010 | HG10-099 | M | 8 | 204 |
| 2010 | HG10-106 | M | 1 | 183 |
| 2010 | HG10-106 | M | 5 | 183 |
| 2010 | HG10-106 | M | 6 | 183 |
| 2010 | HG10-108 | F | 3 | 183 |
| 2010 | HG10-122 | F | 6 | 206 |
| 2010 | HG10-127 | M | 6 | 80 |
| 2010 | HG10-127 | M | 8 | 82 |
| 2010 | HG10-130 | F | 8 | 178 |
| 2010 | HG11-035 | M | 1 | 81 |
| 2010 | HG11-035 | M | 7 | 157 |
| 2010 | HG11-047 | F | 2 | 119 |
| 2010 | HG11-047 | F | 3 | 119 |
| 2010 | HG11-047 | F | 5 | 119 |
| 2010 | HG11-047 | F | 6 | 119 |
| 2010 | HG11-133 | F | 6 | 165 |
| 2010 | M15 | M | 8 | 80 |
| 2011 | 14 | F | 2 | 157 |
| 2011 | F5 | F | 4 | 136 |
| 2011 | F5 | F | 5 | 135 |
| 2011 | F5 | F | 6 | 135 |
| 2011 | F5 | F | 8 | 135 |
| 2011 | F6 | F | 2 | 109 |
| 2011 | HG10-020 | F | 5 | 17 |
| 2011 | HG10-020 | F | 6 | 43 |
| 2011 | HG10-020 | F | 8 | 43 |
| 2011 | HG10-030 | M | 1 | 90 |
| 2011 | HG10-030 | M | 2 | 90 |
| 2011 | HG10-030 | M | 3 | 91 |
| 2011 | HG10-030 | M | 5 | 90 |
| 2011 | HG10-062 | F | 3 | 140 |
| 2011 | HG10-062 | F | 5 | 140 |
| 2011 | HG10-062 | F | 6 | 140 |
| 2011 | HG10-062 | F | 8 | 140 |
| 2011 | HG10-068 | F | 5 | 157 |
| 2011 | HG10-068 | F | 6 | 157 |
| 2011 | HG10-080 | F | 1 | 163 |
| 2011 | HG10-080 | F | 2 | 163 |
| 2011 | HG10-080 | F | 3 | 163 |
| 2011 | HG10-080 | F | 7 | 163 |
| 2011 | HG10-108 | F | 1 | 183 |
| 2011 | HG10-108 | F | 2 | 183 |
| 2011 | HG11-014 | F | 7 | 21 |
| 2011 | HG11-016 | F | 7 | 43 |
| 2011 | HG11-019 | M | 2 | 80 |
| 2011 | HG11-019 | M | 6 | 109 |
| 2011 | HG11-019 | M | 8 | 109 |
| 2011 | HG11-020 | M | 7 | 80 |
| 2011 | HG11-021 | F | 7 | 89 |
| 2011 | HG11-025 | F | 2 | 90 |
| 2011 | HG11-025 | F | 4 | 90 |
| 2011 | HG11-025 | F | 4 | 91 |
| 2011 | HG11-025 | F | 6 | 90 |
| 2011 | HG11-025 | F | 6 | 91 |
| 2011 | HG11-035 | M | 1 | 109 |
| 2011 | HG11-035 | M | 7 | 157 |
| 2011 | HG11-037 | M | 1 | 135 |
| 2011 | HG11-037 | M | 2 | 119 |
| 2011 | HG11-037 | M | 2 | 120 |
| 2011 | HG11-037 | M | 3 | 109 |
| 2011 | HG11-041 | M | 7 | 109 |
| 2011 | HG11-046 | F | 1 | 120 |
| 2011 | HG11-046 | F | 2 | 119 |
| 2011 | HG11-046 | F | 3 | 120 |
| 2011 | HG11-046 | F | 4 | 120 |
| 2011 | HG11-046 | F | 5 | 119 |
| 2011 | HG11-046 | F | 6 | 119 |
| 2011 | HG11-046 | F | 8 | 120 |
| 2011 | HG11-047 | F | 7 | 119 |
| 2011 | HG11-055 | F | 3 | 129 |
| 2011 | HG11-056 | M | 3 | 136 |
| 2011 | HG11-056 | M | 4 | 135 |
| 2011 | HG11-056 | M | 5 | 134 |
| 2011 | HG11-056 | M | 7 | 136 |
| 2011 | HG11-056 | M | 8 | 136 |
| 2011 | HG11-088 | F | 1 | 163 |
| 2011 | HG11-097 | M | 1 | 165 |
| 2011 | HG11-097 | M | 5 | 163 |
| 2011 | HG11-097 | M | 5 | 165 |
| 2011 | HG11-099 | F | 2 | 140 |
| 2011 | HG11-099 | F | 3 | 165 |
| 2011 | HG11-099 | F | 4 | 140 |
| 2011 | HG11-099 | F | 7 | 140 |
| 2011 | HG11-099 | F | 8 | 165 |
| 2011 | HG11-102 | M | 1 | 183 |
| 2011 | HG11-108 | M | 8 | 183 |
| 2011 | HG11-112 | F | 8 | 205 |
| 2011 | HG11-113 | M | 3 | 206 |
| 2011 | HG11-113 | M | 5 | 206 |
| 2011 | HG11-113 | M | 7 | 206 |
| 2011 | HG11-133 | F | 7 | 165 |
| 2011 | HG11-141 | M | 1 | 157 |
| 2011 | M5 | M | 3 | 158 |
| 2011 | M9 | M | 1 | 140 |
| 2012 | 2 | F | 2 | 81 |
| 2012 | 2 | F | 5 | 82 |
| 2012 | 2 | F | 7 | 106 |
| 2012 | 14 | F | 4 | 157 |
| 2012 | F28 | F | 6 | 89 |
| 2012 | F30 | F | 1 | 136 |
| 2012 | F30 | F | 2 | 136 |
| 2012 | F5 | F | 4 | 136 |
| 2012 | HG10-020 | F | 1 | 21 |
| 2012 | HG10-020 | F | 3 | 21 |
| 2012 | HG10-020 | F | 3 | 44 |
| 2012 | HG10-020 | F | 4 | 21 |
| 2012 | HG10-020 | F | 8 | 20 |
| 2012 | HG10-020 | F | 8 | 21 |
| 2012 | HG10-020 | F | 8 | 44 |
| 2012 | HG10-043 | F | 6 | 109 |
| 2012 | HG10-043 | F | 8 | 109 |
| 2012 | HG10-062 | F | 5 | 140 |
| 2012 | HG10-062 | F | 6 | 140 |
| 2012 | HG10-067 | M | 1 | 205 |
| 2012 | HG10-067 | M | 7 | 179 |
| 2012 | HG10-067 | M | 8 | 205 |
| 2012 | HG10-070 | F | 1 | 157 |
| 2012 | HG10-070 | F | 2 | 157 |
| 2012 | HG10-070 | F | 3 | 157 |
| 2012 | HG10-077 | F | 2 | 158 |
| 2012 | HG10-079 | F | 1 | 163 |
| 2012 | HG10-079 | F | 3 | 163 |
| 2012 | HG10-080 | F | 5 | 163 |
| 2012 | HG10-080 | F | 7 | 163 |
| 2012 | HG10-096 | F | 1 | 179 |
| 2012 | HG10-096 | F | 4 | 205 |
| 2012 | HG10-106 | M | 2 | 183 |
| 2012 | HG10-108 | F | 1 | 183 |
| 2012 | HG10-108 | F | 4 | 183 |
| 2012 | HG10-108 | F | 6 | 183 |
| 2012 | HG10-122 | F | 3 | 205 |
| 2012 | HG10-127 | M | 7 | 80 |
| 2012 | HG10-127 | M | 7 | 81 |
| 2012 | HG10-127 | M | 8 | 81 |
| 2012 | HG11-016 | F | 1 | 43 |
| 2012 | HG11-016 | F | 4 | 44 |
| 2012 | HG11-016 | F | 5 | 44 |
| 2012 | HG11-016 | F | 6 | 43 |
| 2012 | HG11-016 | F | 7 | 43 |
| 2012 | HG11-016 | F | 7 | 44 |
| 2012 | HG11-016 | F | 8 | 43 |
| 2012 | HG11-020 | M | 8 | 80 |
| 2012 | HG11-035 | M | 5 | 157 |
| 2012 | HG11-037 | M | 1 | 91 |
| 2012 | HG11-037 | M | 2 | 90 |
| 2012 | HG11-037 | M | 3 | 90 |
| 2012 | HG11-037 | M | 5 | 90 |
| 2012 | HG11-037 | M | 5 | 91 |
| 2012 | HG11-037 | M | 6 | 90 |
| 2012 | HG11-046 | F | 1 | 119 |
| 2012 | HG11-046 | F | 1 | 120 |
| 2012 | HG11-046 | F | 2 | 119 |
| 2012 | HG11-046 | F | 2 | 120 |
| 2012 | HG11-046 | F | 3 | 120 |
| 2012 | HG11-046 | F | 4 | 120 |
| 2012 | HG11-046 | F | 5 | 119 |
| 2012 | HG11-046 | F | 6 | 119 |
| 2012 | HG11-046 | F | 7 | 120 |
| 2012 | HG11-056 | M | 7 | 165 |
| 2012 | HG11-097 | M | 3 | 119 |
| 2012 | HG11-097 | M | 3 | 165 |
| 2012 | HG11-097 | M | 4 | 119 |
| 2012 | HG11-099 | F | 2 | 140 |
| 2012 | HG11-099 | F | 3 | 140 |
| 2012 | HG11-102 | M | 7 | 183 |
| 2012 | HG11-133 | F | 2 | 165 |
| 2012 | HG12-024 | F | 4 | 80 |
| 2012 | HG12-024 | F | 5 | 80 |
| 2012 | HG12-030 | M | 6 | 81 |
| 2012 | HG12-030 | M | 8 | 106 |
| 2012 | HG12-044 | M | 4 | 90 |
| 2012 | HG12-044 | M | 4 | 91 |
| 2012 | HG12-047 | F | 1 | 80 |
| 2012 | HG12-047 | F | 1 | 81 |
| 2012 | HG12-047 | F | 6 | 106 |
| 2012 | HG12-050 | F | 1 | 109 |
| 2012 | HG12-056 | M | 3 | 109 |
| 2012 | HG12-056 | M | 7 | 109 |
| 2012 | HG12-070 | M | 4 | 135 |
| 2012 | HG12-080 | F | 1 | 140 |
| 2012 | HG12-080 | F | 7 | 140 |
| 2012 | HG12-090 | M | 1 | 158 |
| 2012 | HG12-092 | M | 3 | 158 |
| 2012 | HG12-095 | M | 3 | 183 |
| 2012 | HG12-095 | M | 5 | 158 |
| 2012 | HG12-095 | M | 6 | 157 |
| 2012 | HG12-095 | M | 6 | 158 |
| 2012 | HG12-095 | M | 8 | 157 |
| 2012 | HG12-095 | M | 8 | 158 |
| 2012 | HG12-098 | M | 2 | 163 |
| 2012 | HG12-100 | F | 4 | 163 |
| 2012 | HG12-100 | F | 8 | 163 |
| 2012 | HG12-108 | F | 5 | 165 |
| 2012 | HG12-109 | M | 6 | 165 |
| 2012 | HG12-111 | F | 8 | 165 |
| 2012 | HG12-123 | M | 2 | 197 |
| 2012 | HG12-127 | M | 5 | 156 |
| 2012 | HG12-127 | M | 5 | 179 |
| 2012 | HG12-127 | M | 5 | 205 |
| 2012 | HG12-127 | M | 6 | 180 |
| 2012 | M15 | M | 4 | 109 |
| 2012 | M9 | M | 6 | 163 |

Table B. Longitude and Latitude for 46 trap locations where black bear detection events (Table A) occurred in each of 8 occasions during each of 3 sessions (i.e., years). Coordinates are in Universal Transverse Mercator (UTM), zone 17. Trap ID corresponds to Trap in Table A.

| **Trap ID** | **Longitude** | **Latitude** |
| --- | --- | --- |
| 17 | 444984 | 3033338 |
| 18 | 444610 | 3037863 |
| 19 | 445668 | 3039995 |
| 20 | 445048 | 3045404 |
| 21 | 445769 | 3048259 |
| 42 | 448075 | 3032955 |
| 43 | 448588 | 3037349 |
| 44 | 448281 | 3040051 |
| 66 | 453717 | 3028679 |
| 68 | 451173 | 3037410 |
| 79 | 457205 | 2981231 |
| 80 | 456402 | 2984872 |
| 81 | 457525 | 2988614 |
| 82 | 458801 | 2991351 |
| 89 | 457196 | 3021340 |
| 90 | 457572 | 3026385 |
| 91 | 456499 | 3027957 |
| 104 | 461035 | 2981145 |
| 106 | 461719 | 2990132 |
| 109 | 461420 | 3000752 |
| 119 | 459365 | 3042767 |
| 120 | 460585 | 3045929 |
| 121 | 460029 | 3049801 |
| 129 | 464826 | 2980358 |
| 134 | 465388 | 3001332 |
| 135 | 465131 | 3004597 |
| 136 | 466083 | 3008096 |
| 140 | 464073 | 3026369 |
| 146 | 465964 | 3048397 |
| 153 | 470308 | 2978088 |
| 154 | 470358 | 2980072 |
| 156 | 467540 | 2990481 |
| 157 | 468454 | 2993910 |
| 158 | 468994 | 2996768 |
| 163 | 468253 | 3017343 |
| 165 | 468420 | 3025314 |
| 178 | 471317 | 2977831 |
| 179 | 473545 | 2981125 |
| 180 | 473688 | 2984118 |
| 181 | 473500 | 2987733 |
| 183 | 473090 | 2997053 |
| 196 | 473239 | 3048925 |
| 197 | 473595 | 3052772 |
| 204 | 476083 | 2982426 |
| 205 | 476671 | 2985230 |
| 206 | 476506 | 2989144 |
